# Applying Image Recognition and Tracking Methods for Fish Physiology Detection Based on a Visual Sensor

**DOI:** 10.3390/s22155545

**Published:** 2022-07-25

**Authors:** Jia-Ming Liang, Shashank Mishra, Yu-Lin Cheng

**Affiliations:** 1 Department of Electrical Engineering, National University of Tainan, Tainan 70005, Taiwan; d10982003@stumail.nutn.edu.tw; 2 Department of Computer Science and Information Engineering, Chang Gung University, Taoyuan 33302, Taiwan; m0829013@cgu.edu.tw

**Keywords:** image recognition, object tracking, correction mechanism, Internet of Things

## Abstract

The proportion of pet keeping has increased significantly. According to the survey results of Business Next, the proportion of Taiwan families keeping pets was 70% in 2020. Among them, the total number of fish pets was close to 33% of the overall pet proportion. Therefore, aquarium pets have become indispensable companions for families. At present, many studies have discussed intelligent aquarium systems. Through image recognition based on visual sensors, we may be able to detect and interpret the physiological status of the fish according to their physiological appearance. In this way, it can help to notify the owner as soon as possible to treat the fish or isolate them individually, so as to avoid the spread of infection. However, most aquarium pets are kept in groups. Traditional image recognition technologies often fail to recognize each fish’s physiological states precisely because of fish swimming behaviors, such as grouping swimming, shading with each other, flipping over, and so on. In view of this, this paper tries to address such problems and then proposes a practical scheme, which includes three phases. Specifically, the first phase tries to enhance the image recognition model for small features based on the prioritizing rules, thus improving the instant recognition capability. Then, the second phase exploits a designed fish-ID tracking mechanism and analyzes the physiological state of the same fish-ID through coherent frames, which can avoid temporal misidentification. Finally, the third phase leverages a fish-ID correction mechanism, which can detect and correct their IDs periodically and dynamically to avoid tracking confusion, and thus potentially improve the recognition accuracy. According to the experiment results, it was verified that our scheme has better recognition performance. The best accuracy and correctness ratio can reach up to 94.9% and 92.67%, which are improved at least 8.41% and 26.95%, respectively, as compared with the existing schemes.

## 1. Introduction

With the decrease in the total fertility rate (TFR), more and more people keep pets to obtain spiritual companionship and reduce the sense of isolation in modern society. In Taiwan, fish pets are very common in families. According to the survey results of Business Next [1], the amount of fish pets in Taiwan approaches 33% of the overall proportion, as keeping fish has many benefits, such as reducing life stress, improving mental health, and even decreasing disruptive behaviors and increasing appetites for people with Alzheimer’s disease [2,3].

However, raising fish requires much patience and requires keeping watch carefully, such as daily regular and quantitative feeding and observing the fish physiological variations and environmental changes, so as to avoid the spread of disease. Although traditional intelligent aquarium systems (e.g., [4,5]) can automatically feed and adjust the water temperature and/or lighting functions, they cannot immediately inform the owner about the physiological variation of fish in the aquarium, especially for diseased or dead fish. With the failure to separate or remove these diseased or dead fish in a timely manner, more fish will die soon owing to the spread of infection.

In the literature, existing studies may propose and use image processing techniques to identify fish species or the aquarium status, such as converting color images into gray-scale images for image analysis [6], increasing the number of images based on image synthesis [7], rotating and cropping image features to increase the total training data [8,9], or using neural networks to improve image recognition [10,11]. However, these studies identify fish only by a single image, which may incur misidentification owing to fish swimming behaviors, such as grouping, shading, and flipping over, and may potentially limit the recognition performance.

Therefore, this paper studies how to effectively and precisely detect the physiological variation of fish under the consideration of fish behaviors. We choose guppy (*Poecilia reticulata* [12]) as a case study, which is the most common fish pet in aquariums [13]. Then, we propose an effective fish physiological detection scheme by integrating image recognition with a multi-object tracking mechanism for identification. The main idea is to analyze the results of consecutive images and combine the image recognition results with the multi-target tracking mechanism to accurately detect the fish physiological status. In this way, it can effectively reduce the misidentification of fish physiological status and thus achieve higher accuracy. In addition, the proposed tracking mechanism can also track the physiological states of certain fish, so as to take care of special fish pets. Therefore, a fish-ID correction mechanism is designed. That is, during the process of fish physiology detection, the correction mechanism will be activated periodically to achieve accurate tracking. In the simulations, the experimental results will show that the proposed scheme has better performance in terms of precision (89.8%), recall (95.02%), F1-score (92.4%), and accuracy (94.9%).

The rest of this paper is organized as follows. Related work is discussed in Section 2. Section 3 presents the problem definition. Section 4 presents our scheme. The experiment results are shown in Section 5. Section 6 concludes the paper.

## 2. Related Work

In the literature, studies [6,7,8,9,14,15,16] have discussed how to use image recognition to identify the species and state of aquariums. Specifically, the work of [6] proposes to convert color images into gray-scale images to form a gray-scale histogram, and then segment the gray-scale histogram and extract features to use principal component analysis. Thus, it can reduce the amount of feature extractions for fish species’ identification. The work of [7] tries to convert a large number of images of various fish in different natural environments through image synthesis, in order to solve the problem of species data lacking in the CNN identification model. The work of [8] uses the VGG16 network to classify fish, and enhances the number of features of the original image by rotating, cropping, and scaling to increase the amount of training image data. The study of [9] exploits YOLOv1 (You Only Look Once) to distinguish different species of fish in real time, and reduces the misclassification rate of bottom-dwelling fish by randomly cropping images of various seabeds. The authors of [14] use a variety of networks for classification and adopt an elastic rotation-based data augmentation method to avoid overfitting caused by imbalanced training data sets. The work of [15] leverages deep learning-based object detection systems such as YOLOv2 and SSD [16] (Single Shot MultiBox Detector), and proposes using contextual information of surrounding scenes to improve the overall recognition accuracy.

In addition, the studies of [10,11,17,18] try to improve existing neural networks to reduce overfitting and solve the problems of image recognition. Specifically, the study of [10] proposes to connect the layer of spatial pyramid pooling (SPP) before the fully-connected layer of a dense neural network to realize multi-size input of images and reduce network size. The work of [11] proposes to add more convolutional layers to the basic architecture of VGG16, and performs transfer learning on various other network models to enhance the feature extraction capability in the training process. The study of [17] designs a new set of CNNs consisting of three branches that classify fish species. The study of [18] proposes a special cross-layer pooling algorithm, which combines the features from different layers of the deep neural network and passes them to the SVM for classification. In this way, it can reduce the amount of training data. However, all of the above studies classify and identify fish based on the single image, which potentially limits the recognition performance. Therefore, this work will consider the above issue.

## 3. Problem Definition

**Figure 1:**
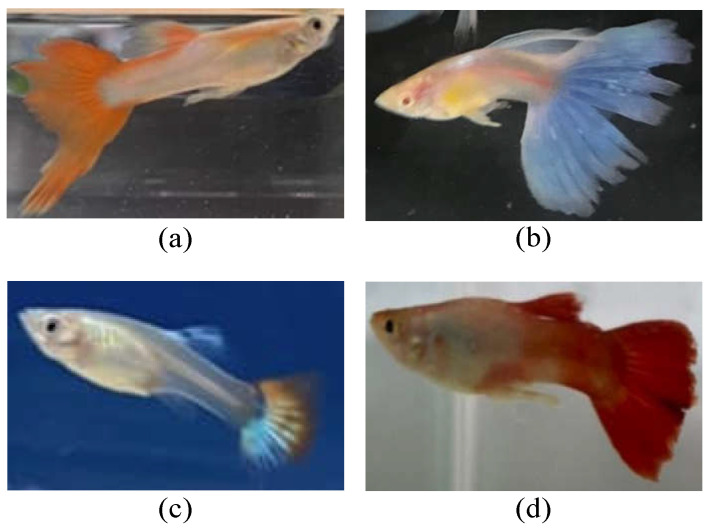
Illustration of guppy fish (**a**–**d**).

In this paper, we consider the guppy fish (scientific name: *Poecilia reticulata* [12], which is the most common fish species in aquariums [13]) as the case study to recognize the physiological state of fish, as shown in Figure 1.

**Figure 2:**
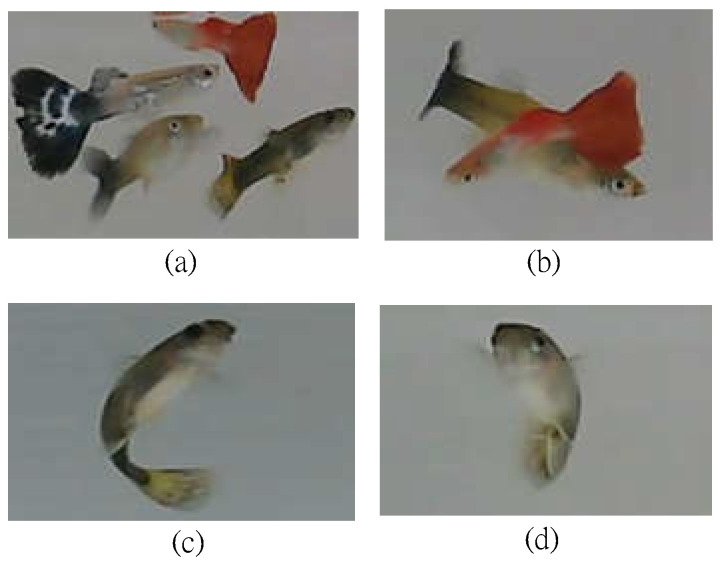
Illustration of fish swimming behaviors: (**a**) grouping swimming, (**b**) shading with each other, (**c**) flipping body, and (**d**) turning over for breath.

Specifically, we consider an aquarium space *L* with length (*l*), width (*w*), and height (*h*) in cm. We assume that *N* guppies swim freely in the aquarium space *L*, where each fish has its own ID (i.e., name), denoted by *ID_i_, i* = 1…*N*. Here, we consider the most common physiological states of guppies [19], including *healthy* (marked as *H*), *diseased* (rotten tail, marked as *R*), and *dead* (marked as *D*). In addition, we also consider fish swimming behaviors, including group swimming, shading with each other, flipping body, and turning over for breath, among others, as shown in Figure 2.

Here, we assume a total of *T* image frames were captured by the visual sensor over the aquarium space, and parts of the frame *f =* 1…*m* (*m < T*) were labeled as the dataset <*B_f_, I_f_, S _f_*>, as shown in Table 1, where *B_f_, I_f_*, and *S_f_* represent the list of labeled bounding boxes, labeled fish-IDs, and the labeled physiological states of fish in frame *f*, respectively. In addition, each bounding box has a center coordinate (*x_i_, y_i_*) and length (*l_i_*) and width (*w_i_*), e.g., *B_f_ = ((x_1_, y_1_, l_1_, w_1_), (x_2_, y_2_, l_2_, w_2_), (x_3_, y_3_, l_3_, w_3_),…, (x_i_, y_i_, l_i_, w_i_))*, where these bounding boxes are labeled by the fish’s name, i.e., *I_f_ = (ID_1_, ID_2_, ID_3_, …, ID_i_)*, and labeled by physiological states, i.e., *S_f_ = (s_1_, s_2_, s_3_, …, s_i_)*, where *s_i_* ∈{*H, R, D*}, respectively. On the other hand, there are unlabeled data lists <*B_x_, I_x_, S_x_*>, *x = (m +* 1*)*…*T*, where they are the unknown list of bounding box coordinates, unknown fish-IDs, and unknown physiological states, respectively. Our aim is to address the following problem: How to correctly identify the unknown data list <*B_x_, I_x_, S_x_*> *, x = (m +* 1*)*…*T* through the labeled data list <*B_f_, I_f_, S_f_*>, *f =* 1…*m*.

## 4. The Proposed Scheme

**Figure 3:**
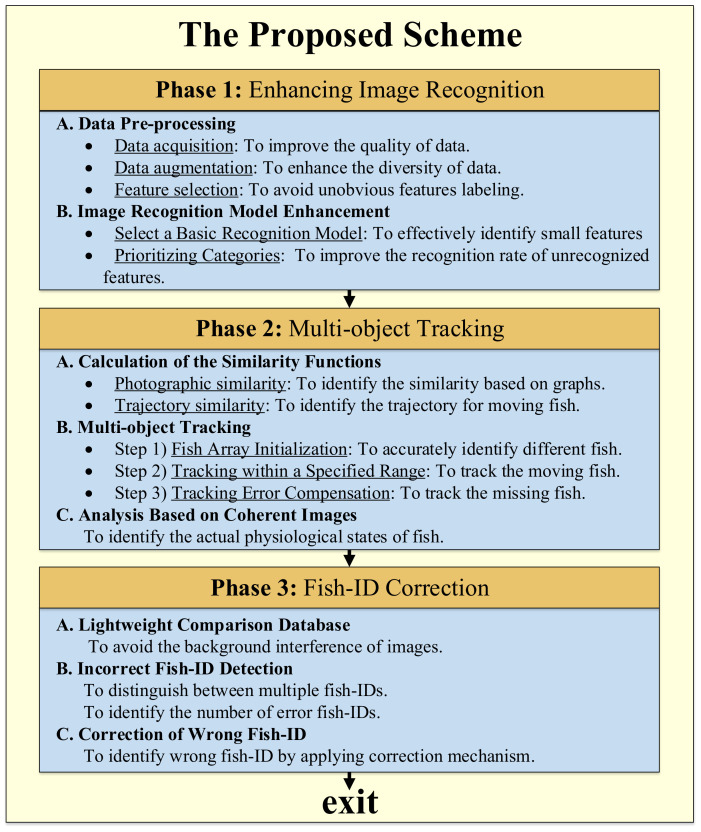
The concept of the proposed scheme.

To precisely detect the fish physiological states, we propose a practical scheme including three phases, as shown in Figure 3. The first phase is to perform data pre-processing, including data acquisition, data augmentation, and feature selection, to obtain high-quality training data. In addition, we select and enhance an image recognition model to improve the detection accuracy based on the prioritizing rules, especially for the small-scale features. The second phase is to design a multi-object tracking mechanism based on the calculation of similarity functions according to the fish’s photograph and trajectory, and then analyze the tracked fish over consecutive frames to determine their actual physiological states to improve recognition accuracy. The third phase is to develop a fish-ID correction mechanism. We build a comparison lightweight database for fish images based on the histogram coincidence to estimate when to trigger the correction mechanism and dynamically perform correction. The detailed steps are depicted as follows.

### 4.1. Phase 1: Enhancing Image Recognition

The goal of Phase 1 is to perform data pre-processing and enhancing the image recognition model for small feature recognition.

**A.**  **Data Pre-processing**

In order to obtain more high-quality data, the data pre-processing is realized by three stages, includes data acquisition, data augmentation, and feature selection.

**Figure 4:**
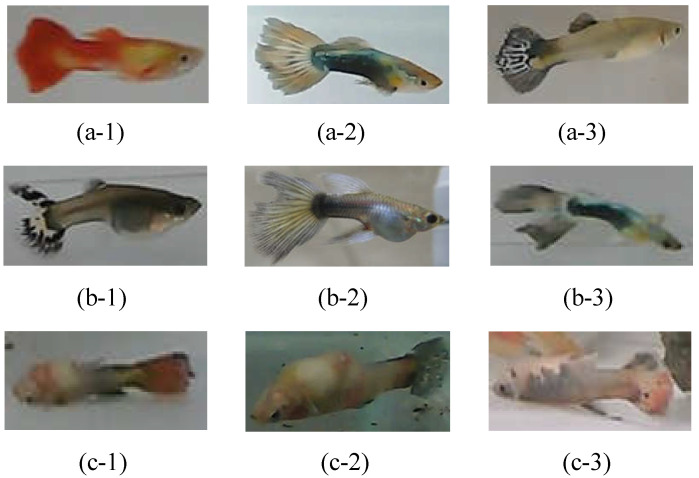
Three physiological states of guppies: (**a**) healthy, (**b**) rotten-tail, and (**c**) dead.

**Figure 5:**
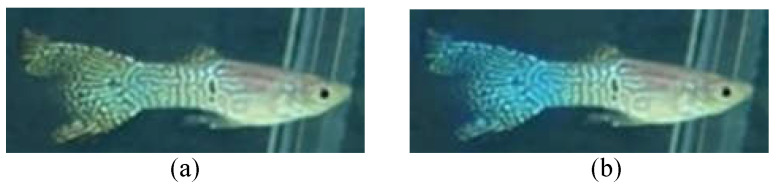
With random discoloration to improve data diversity: (**a**) before dyeing and (**b**) after dyeing.

**Figure 6:**
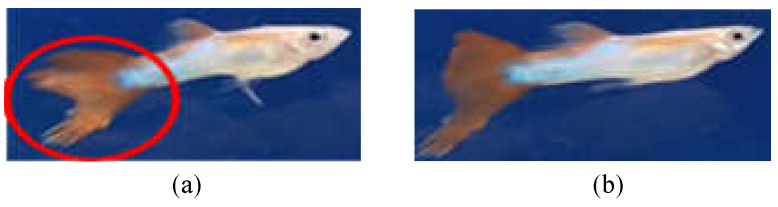
Features of rotten tail: (**a**) rotten tail is obvious and (**b**) rotten tail is not obvious.

- **Data acquisition:** We collected more than 6000 pictures of guppies, including three physiological states: health, disease (rotten tail), and death, as shown in Figure 4. These pictures are obtained from (1) online guppy photos and (2) the real guppy photographed by the visual sensors (cameras). Note that the online guppy photos are from Taiwan Fish Database [20], Guppy Dataset [21], and other searching engine results; the real guppy pictures are photographed by the visual sensors with a pixel resolution of 4032 × 3024 and 5184 × 3880, respectively. The characteristics of the guppy pictures include their various appearances, such as front view, back view, side view, swimming up, and swimming down, under the environments of clear background and background with landscaping.

- **Data augmentation:** To improve the overall data diversity, we randomly select photos to process with discoloration, rotation, or mirroring to increase the amount of training data, which can make the recognition model more general and avoid overfitting [8,22,23]. As the images of fish with certain features may not be easy to obtain (such as rotten tails), we also dye the existing rotten-tail images with random colors, such as blue, green, purple, red, and black colors, which are the common colors of guppy tails, in order to balance the data of the three physiological states, as shown in Figure 5. This can potentially improve the recognition capability.

- **Feature selection**: According to the literature [24,25], the caudal fin of rotten-tail fish will have irregular notches or a jagged appearance. Thus, we label these features according to the above description and consult the experts working in the aquarium for guidance [26]. Sometimes, it is difficult to judge because of the irregular swimming of the fish and the angle of view, as shown in Figure 6. Therefore, we only can label the feature of the images that meet the above description, so as to avoid labeling with inconspicuous characteristics of rotten tails. Note that if the images of fish with inconspicuous features are used as training data, the tails of healthy fish are easily misidentified as rotten-tail fish, potentially resulting in a decrease in the recognition rate.

**B.**  **Enhancing the Image Recognition Model**

**Figure 7:**
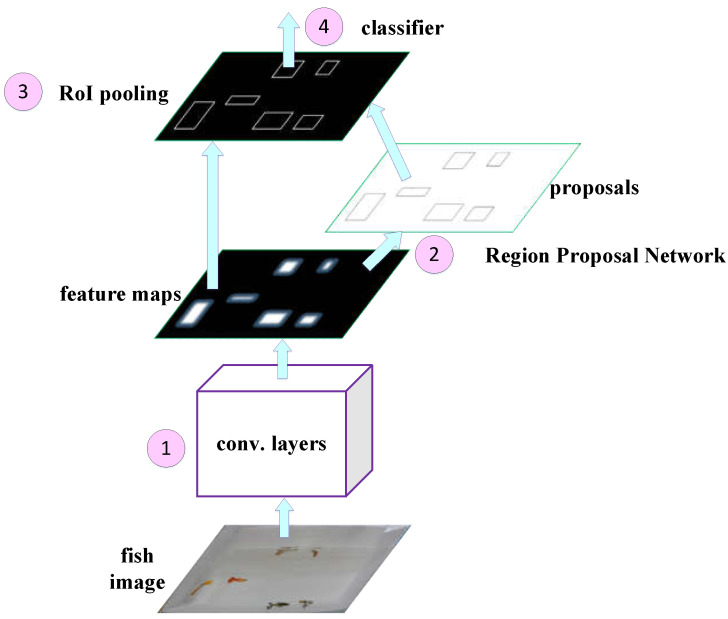
The architecture of the faster R-CNN model.

In order to effectively identify small features (e.g., rotten tail), we adopt faster R-CNN [27] as the basic model for image recognition, as shown in Figure 7, which has the following features:

1. The two-stage detection method is adopted to separate the object position detection and the object identification, so that it has a better performance in terms of accuracy. Compared with the one-stage detection method, the detection of small targets will be more accurate.
2. It is more flexible and does not have too many restrictions on image input. Images with different aspect ratios are allowed, and images with different aspect ratios can also be used for training.
3. The RPN (region proposal network) is exploited as the extraction network of the candidate frame, which improves the speed while improving the accuracy.

Specifically, the faster R-CNN model is depicted as follows.

**Figure 8:**
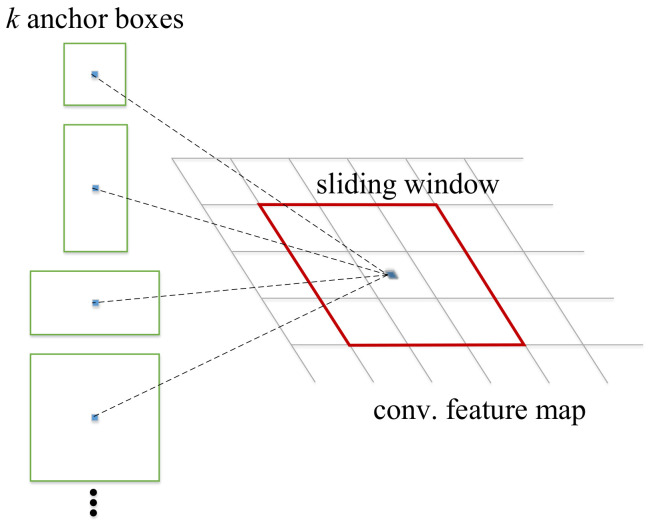
Illustration of anchor boxes.

Step (1)  First, it extracts the feature map of the image with the pre-trained network (e.g., VGG16, ResNet), and the feature map will be shared by the subsequent RPN layer and the fully connected layer. Note that we use VGG16 as our pre-trained network, which has a better performance in small feature recognition.
Step (2)  Then, the RPN layer generates candidate boxes (region of interest, RoI). This layer replaces the selective search used by the previous versions of R-CNN and faster R-CNN to extract candidate boxes, and leverages the concept of anchor boxes by the operation of sliding window on the feature map. The position of the center point of the current sliding window corresponds to the pixel space of the original image, and then k anchor boxes with different sizes and different aspect ratios are generated on the original image. The size of each anchor box will vary according to the size of the input image, as shown in Figure 8. Finally, it estimates whether the anchor boxes belong to the target (positive) or not (negative) through the softmax function, and then uses the bounding box regression to adjust the position information of the anchor boxes to obtain the accurate position of candidate frames.
Step (3)  After that, RoI pooling is performed on the previously generated feature map and the candidate frame generated by the RPN layer. As the subsequent classification layer requires an input of the same size, RoI pooling maps candidate boxes of different sizes to the feature map and makes them the same size, and then passes the features of the same size to the subsequent classification layer for classification.
Step (4)  Finally, the classification layer calculates the probability that the object in each candidate frame belongs to each category through the fully connected layer and softmax, and then determines to which category the object belongs, and uses bounding box regression again to obtain a more accurate target detection frame.

**Figure 9:**
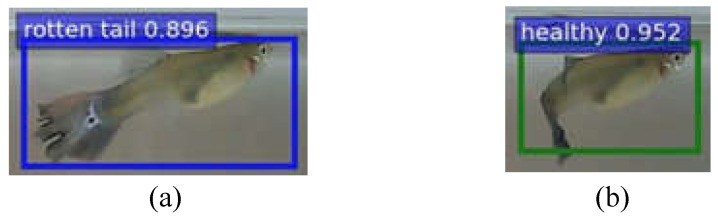
Fish flips over would easily lead to misidentification: (**a**) a rotten-tail fish and (**b**) misidentified as a healthy fish when turning around.

- **Prioritizing Categories**
  1. To improve the recognition rate of unrecognized features, we set the recognition priority for each category and adjust the confidence thresholds of different categories.
  2. The identification priority is also set by the following order: dead > diseased (rotten-tail) > healthy. As the diseased (rotten-tail) fish would be recognized as healthy from certain angles, as illustrated in Figure 9, the higher priority should be given for the diseased (rotten-tail) category to avoid recognizing them as healthy. Note that the characteristics of fish death are the most obvious, so the priority is the highest.
  3. Next, let the threshold of the health category be $t_{H}^{\sim }$, the threshold of the diseased (rotten-tail) category be $t_{R}^{\sim }$, and the threshold of the death category be $t_{D}^{\sim }$. Then, according to the above priority, when the confidence value of the category is greater than the corresponding threshold (e.g., $t_{H}^{\sim }$, $t_{R}^{\sim }$, $t_{D}^{\sim }$), it will be determined as the corresponding category.

Through the above operations, the identification results of each category can be more accurate.

### 4.2. Phase 2: Multi-Object Tracking

The objective of Phase 2 is to track each fish’s ID through continuous images and identify the fish physiological state over coherent images, and finally estimate the fish’s actual physiological state to improve the recognition accuracy. Specifically, we define a similarity function of the bounding boxes of two recognition frames at the previous time (*t* − 1) and the current time (*t*). Then, we calculate the similarity function in terms of photograph similarity and trajectory similarity, and track the fish-ID within the possible range of activities; finally, continuous observation is carried out for a period of time and then the actual physiological state of the fish is determined. The detailed steps are described as follows:

**A.**  **Calculation of the Similarity Functions**

First, we let $B_{k}\left(\tau \right)$ represent the similarity of the two bounding boxes in the previous and current images, where *τ* represents the time when starting and ending, and *k* represents the bounding box pair to be compared in the two images. $B_{k}\left(\tau \right)$ is defined as the product of the photographic similarity $I_{k}^{S}\left(\tau \right)$ and the trajectory similarity $C_{k}^{S}\left(\tau \right)$, i.e.,

$$B_{k}\left(\tau \right)=I_{k}^{S}\left(\tau \right)\times C_{k}^{S}\left(\tau \right).$$

- **Photographic similarity** $I_{k}^{S}\left(\tau \right)$

The photographic similarity $I_{k}^{S}\left(\tau \right)$ is defined by the multiplication of the string similarity $S_{k}^{str}\left(\tau \right)$ and the distance difference of center coordinate movement $D_{k}^{norm_{diff}}\left(\tau \right)$, i.e.,

$$I_{k}^{S}\left(\tau \right)=S_{k}^{str}\left(\tau \right)\times D_{k}^{norm_{diff}}\left(\tau \right).$$

Note that $S_{k}^{str}\left(\tau \right)=1-\frac{L_{k}\left(\tau \right)}{A_{k}\left(\tau \right)}$ is the string similarity, where $L_{k}\left(\tau \right)$ is the image of bounding box recognized before and after through the Hash algorithm [28,29], which calculates the edit distance (*Levenshtein distance*) based on a set of binary strings [30,31]. In addition, $A_{k}\left(\tau \right)$ is the longer distance of the two-image pair (before and after), which is used to normalize the value of $L_{k}\left(\tau \right)$. Thus, the value of $S_{k}^{str}\left(\tau \right)$ is between 0 and 1.

**Figure 10:**
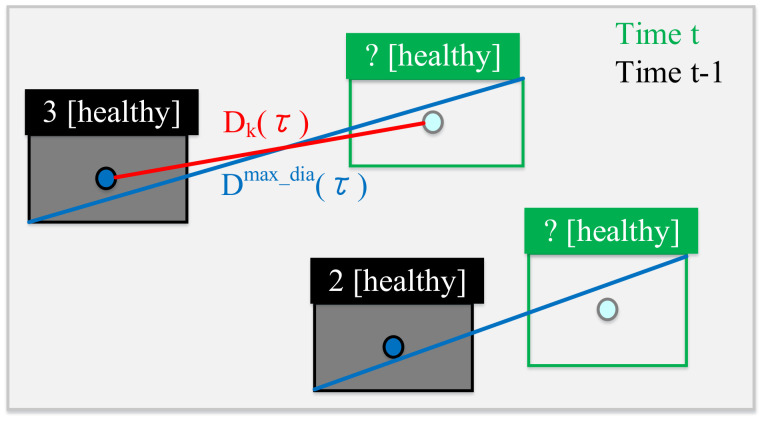
Illustration of $D_{k}\left(\tau \right)$ and $D^{max\_dia}\left(\tau \right).$

$D_{k}^{norm_{diff}}\left(\tau \right)=1-\frac{D_{k}\left(\tau \right)}{D^{max\_dia}\left(\tau \right)}$ is the normalization of the distance between the center coordinates of the bounding box (before and after), as shown in Figure 10, where $D_{k}\left(\tau \right)$ is the distance between the center coordinates of the bounding box (before and after), and $D^{max\_dia}\left(\tau \right)$ is the largest diagonal line between the two bounding boxes.

- **Trajectory similarity** $C_{k}^{S}\left(\tau \right)$

Trajectory similarity $C_{k}^{S}\left(\tau \right)$ is defined by the cosine operation of the two fish movements and normalized by a coefficient α, i.e.,

$$C_{k}^{S}\left(\tau \right)=C_{k}^{norm}\left(\tau \right)\times \alpha +\left(1-\alpha \right)$$

where $C_{k}^{norm}\left(\tau \right)=\frac{\;(\tau )+1}{2}(0<C_{k}^{norm}\left(\tau \right)<1)$ is the normalized value of the similarity of the fish moving (before and after), where $C_{k}\left(\tau \right)$ is defined as follows:

$$C_{k}\left(\tau \right)=cosine\left(V\left(\tau^{\prime }\right),\;V\left(\tau \right)\right)$$

**Figure 11:**
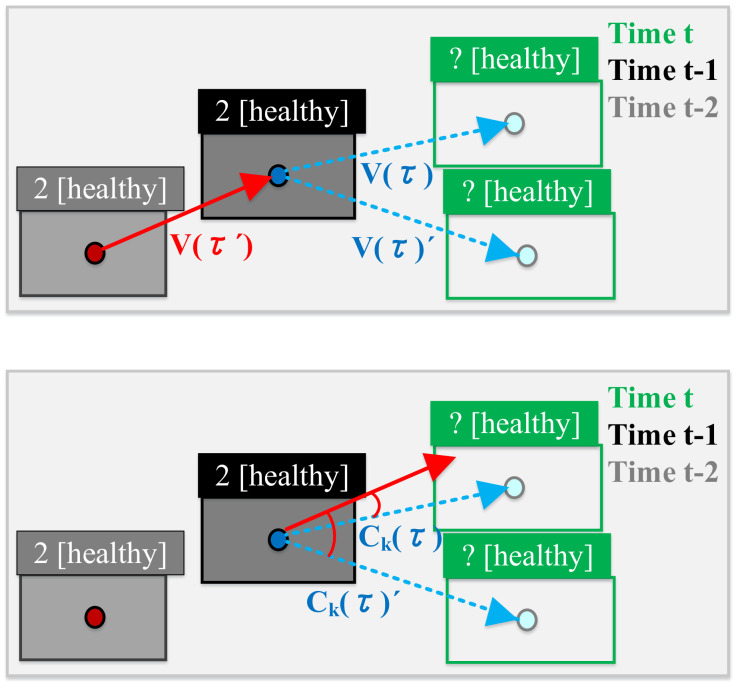
Illustration of $V\left(\tau^{\prime }\right)$ and $V\left(\tau \right)$.

Note that *τ’* represents the time before the previous moment (*t* – 2) and the previous moment (*t* − 1), so $V\left(\tau^{\prime }\right)$ and $V\left(\tau \right)$ are the moving vectors of previous moment and the current moment, i.e., $V\left(\tau^{\prime }\right)=vector\left(t-2,\;t-1\right)$ and $V\left(\tau \right)=vector\left(t-1,\;t\right)$, respectively. This can be illustrated in Figure 11.

Here, the value of $C_{k}\left(\tau \right)$ is between –1 and 1, because we assume that the swimming direction of the fish might not change dramatically under normal circumstances. Thus, when the value is closer to 1, it means that the trajectory vectors between the two bounding boxes are more similar, which is more likely the same fish. Note that α is a coefficient ranging from 0 to 1, which is used to adjust $C_{k}^{norm}\left(\tau \right)$ so that $C_{k}^{S}\left(\tau \right)$ is always positive and within the range of $\left(1-\alpha \right)<C_{k}\left(\tau \right)<1$. This can help us to control the impact of fish swimming on the overall similarity.

Through the above calculation, the final bounding box similarity $B_{k}\left(\tau \right)$ can be obtained.

**B.**  **Multi-object Tracking Design**

In the following, the similarity of two bounding boxes defined by previous subsection will be leveraged and the relevant information will used for similarity comparison. The detailed steps are as follows.

Step (1)  **Fish Array Initialization**: First, we create a fish array *A = [ID_i_, s_i_, (x_i_, y_i_, w_i_, h_i_)], i = 1..N* to store their IDs (initially a random number), state, and bounding box coordinate of each fish, which is identified by faster R-CNN in Phase 1.
Step (2)  **Tracking within a Specified Range**: Next, we take the center coordinate of the bounding box at the previous time (*t* − 1), select a specific range at the radius $r^{\sim }$, and search for the center coordinates of all the bounding boxes at the current time (*t*) within this range. Then, we compare the bounding box of the previous moment (*t* − 1) with all of the identification frames of the current time (*t*) within the circle selection range and calculate their similarity by $B_{k}\left(\tau \right)$. After the comparison, the bounding box with the highest similarity at the previous time (*t* − 1) is selected, which will inherit the ID of the former time, and replace it with the initial ID, and then update the tracking array accordingly.
Step (3) **Tracking Error Compensation**: In the following tracking process, if there are fish that fail to be detected, or the bounding box is missing because of shading with each other, we design a tracking error compensation mechanism to record the relevant information at the moment (*t* − 1) before the ID is missing. Once the ID tracking is performed at the next moment (*t* + 1), the range of the search radius $r^{\sim }$ is expanded by Ω times for ID exploration, so as to avoid the tracking failure caused by the fish moving far away. Note that when the system is started, the number of fish will be matched to the number of bounding boxes. If the fish disappears over $b^{\sim }$ frames, the current missing ID will be stored. Once the fish appears again, the missing ID of the fish will be assigned.

**C.**  **Analysis Based on Coherent Images**

To accurately identify the physiological state of the fish, especially for the smaller feature (i.e., rotten tail), we propose to observe consecutive frames ($\tilde{f_{t}}$) to track and calculate the actual physiological status of the fish with the same ID *i*. That is, all categories identified before a period of frames from current time *t* (i.e., *f =* (*t−*$\tilde{f_{t}}$)...*t*) are calculated according to the “OR” (union) operation, i.e.,

$$s_{i}={⋃}_{f=t-\tilde{f_{t}}}^{f=t}{\hat{s}}_{f,i}.$$

Note that ${\hat{s}}_{f,i}$ is the physiological state of the fish with ID *i* in frame *f*. The rotten-tail category is marked by 

${\hat{s}}_{f,i}=$1 and the health category is marked by ${\hat{s}}_{f,i}=0$. After the calculation, the actual category of the fish is confirmed. As “1” has a decisive factor in the “OR” operation and the rotten-tail feature is less obvious than the healthy feature, the operation of taking the rotten-tail state as “1” can improve the recognition rate of such a category.

### 4.3. Phase 3: Fish-ID Correction

The objective of Phase 3 is to detect and track incorrect fish-ID images and correct them. Specifically, this phase is divided into three steps. First, we establish a temporary comparison database of fish images; then, we perform image preprocessing for each fish in the database to simplify the fish features; through the comparison of the histogram coincidence of consecutive images, the fish-ID is tracked incorrectly will be found; finally, the image with the wrong ID is cross-matched accordingly.

In the following, we use *i* to denote the current fish-ID, and *P(i)*, *Q(i)*, and *R(i)* are denoted by the fish images with incorrect ID, the ID of fish images in the comparison database, and the target fish-ID to be checked, respectively. The detailed steps are as follows.

**Figure 12:**
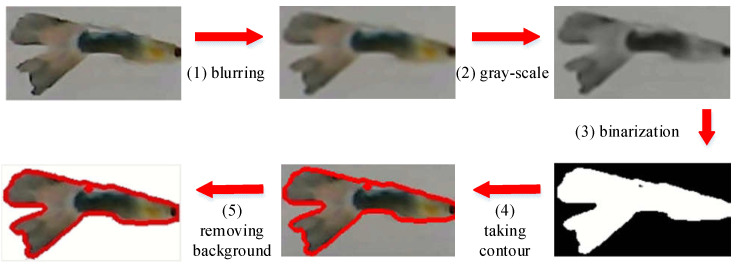
Illustration of fish image pre-processing.

**A.**  **Establishment of a Lightweight Comparison Database**: According to the ID within $\tilde{m}$ frames after the system starts, each fish is intercepted in *λ* valid images and added to the fish comparison database. Then, we expand these images through mirroring and flipping [8]. After that, we have 2λ images for each fish-ID *i*, denoted by *Q(i)*. Here, to avoid the background interference of fish images, we perform the following image processing processes [32], including (1) blurring, (2) gray-scale, (3) binarization (black and white), (4) taking contours, (5) removing background, and then storing the images in Q(i), as illustrated in Figure 12. In this way, the interference caused by the background can be effectively reduced.
**B.**  **Incorrect Fish-ID Detection**
  Step (1)  For the fish images stored in *Q(i)*, the BGR histogram of *R(i)* is generated by the OpenCV function (calcHist).
  Step (2)  Then, we use the function (compareHist) to perform a histogram coincidence comparison between the image in the database *Q(i)* and the target image *R(i)*.
  Step (3)  Next, we compare the target image *R(i)* with the λ images in *Q(i)* and calculate the average value based on the histogram gravity and degree *H(i)*. If the calculated result exceeds the pre-defined threshold (${\tilde{t}}_{H}$), it means that the image similarity of the target ID is low.
  Step (4)  To avoid a false alarm caused by temporary low similarity such as fish turning around or shading with each other, it will observe the similarity through $\tilde{f_{H}}$ frames. Once the consecutive $\tilde{f_{H}}$ frames all have low similarity, it is identified as a wrong ID.
**C.**  **Correction of Wrong Fish-ID**
  1. The above incorrect fish-ID detection will be activated periodically by $p^{\sim }$ frames. Once it detects at least two wrong IDs, it will activate the correction mechanism.
  2. Assuming that the total number of wrong IDs is Φ ≥ 2, the correction mechanism will cross-match all the wrong IDs’ images in the comparison database *Q(i)*.
  3. If the IDs of the two most similar images are matched, the wrong IDs will be updated by the ID in the comparison database. The operation will be executed continuously until the number of wrong IDs is less than 2.

Note that if the period of incorrect fish-ID detection is shorter, the ID correction rate can be increased. Contrarily, if the detection period is longer, the system loading for the correction can be decreased. The period can be set depending on the requirements.

Through the above operations, the fish-ID can be corrected in a timely manner to ensure the correctness.

## 5. Performance Evaluation

In this section, we conduct real experiments under an aquarium space *L =* 35 *×* 18 *×* 11 (cm). We collected 6000+ images of guppy fish, including images of healthy (2000+), disease/rotten-tail (2000+), and dead (2000+), for labeling and testing. We compare our scheme against five schemes, including YOLOv4 [33], YOLOv5 [34], SSD [16], and the two original faster R-CNNs [27]. Specifically, YOLOv4 and YOLOv5 are well-known image recognition models with fast object detection, which perform better than the previous versions (i.e., YOLOv1 [9] and YOLOv2 [15] in the literature). SSD can detect objects through large feature maps with high speed and better accuracy. The original faster R-CNN methods, including two common models (i.e., ResNet101 (denoted by RES101) and VGG16), are used as the control group to show the improvements and differences from ours. Note that the common parameters of all schemes are shown in Table 2. All of the parameters are chosen by referring to the suggestion of the original references [16,27,33,34] and have been fine-tuned based on the hardware restriction to achieve well-performance [35,36].

For physiological state identification, we evaluate the performance of the proposed methods by the following performance metrics based on the confusion matrix, as shown in Table 3. They are precision, recall, F1-socre, and accuracy, where precision = $\frac{\mathrm{TP}}{\mathrm{TP}+\mathrm{FP}}$, recall = $\frac{\mathrm{TP}}{\mathrm{TP}+\mathrm{FN}}$, F1-score = $\frac{\left({1+\beta }^{2}\right)\times \mathrm{Precision}\times \mathrm{Recall}}{\beta^{2}\left(\mathrm{Precision}+\mathrm{Recall}\right)}$, and accuracy = $\frac{\mathrm{TP}+\mathrm{TN}}{\mathrm{TP}+\mathrm{TN}+\mathrm{FP}+\mathrm{FN}}$.

### 5.1. Precision

**Figure 13:**
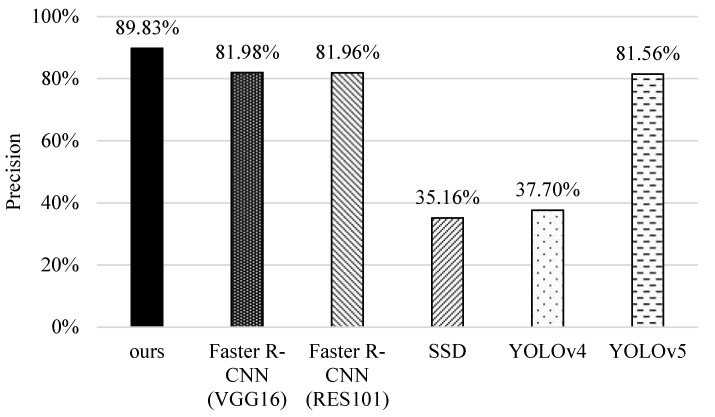
Comparisons of the precision of different methods.

First, we investigate the performance of different schemes in precision. As shown in Figure 13, SSD and YOLOv4 have poor performance. As they are one-stage detection methods, only a single neural network is used to detect object and classify objects. Note that YOLOv5 enhances the detector, including the modules of input, backbone, neck, and dese prediction; thus, it can detect more bounding boxes and performs better than them. The original faster R-CNNs perform well because they use a two-stage detection with RPN to find candidate regions of objects and then classify and correct these regions to improve the recognition results of small features. Our method performs the best. This is because our method identifies different categories based on a predefined priority order, and then analyzes categories through coherent images by a multi-object tracking mechanism, thus improving the overall performance.

### 5.2. Recall

**Figure 14:**
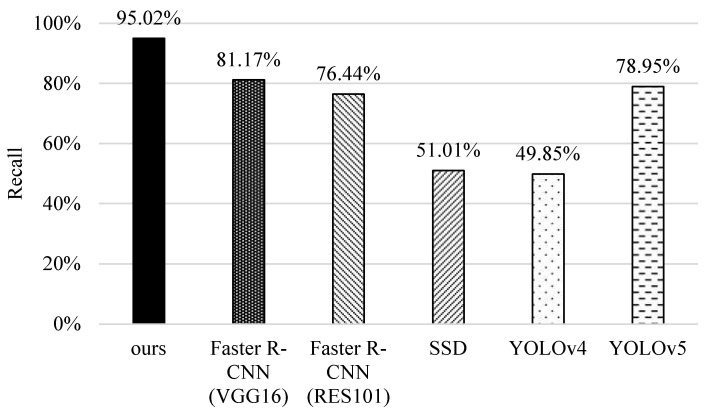
Comparisons of recall of different methods.

Next, we investigate the performance of different methods on recall. As shown in Figure 14, the recall value of SSD and YOLOv4 is lower because of the use of one-stage detection. They are difficult to distinguish between the rotten-tail and healthy fish and even fail to identify the bounding box. YOLOv5 performs better because it improves the detection process and involves more coefficients to control the network layers and channels for object identification. The original faster R-CNNs perform better because of better recognition of small objects. Specifically, RES101 performs worse than VGG16 because RES101 adopts a pooling operation after the first convolutional layer, which causes some features to be lost. It is worth noting that our scheme performs the best because it leverages the prioritizing classification and analyzes coherent images based on the proposed tracking mechanism, which can avoid misidentification in specific categories, such as rotten-tail and healthy ones.

### 5.3. F1-Score

**Figure 15:**
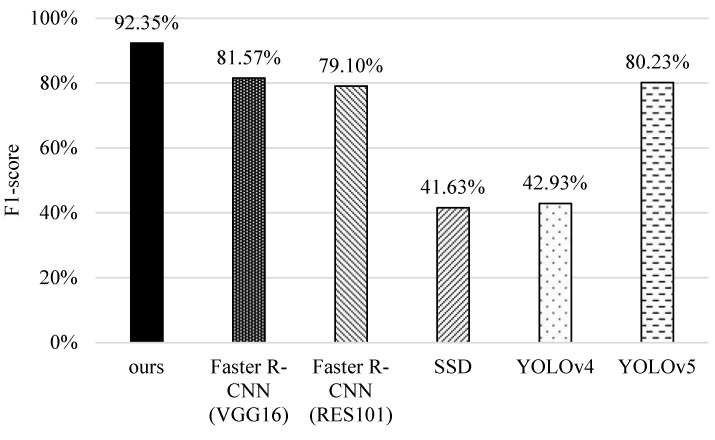
Comparisons of F1-scores of different methods.

Furthermore, we compare the F1-score of different schemes in Figure 15. Similarly, SSD and YOLOv4 have lower F1-score values owing to their misidentification of small features. YOLOv5 performs better because it enhances the input layer with data argumentation, thus improving the identification capability. The original faster R-CNNs are better because of their recognition ability of small objects. It is worth noting that the proposed scheme outperforms other schemes. The reason is that it enhances the original faster R-CNN based on the prioritizing classification with proper confidence thresholds, and combines image recognition with tracking concepts to estimate the results by coherent images; thus, it has better performance in terms of F1-score.

### 5.4. Accuracy

**Figure 16:**
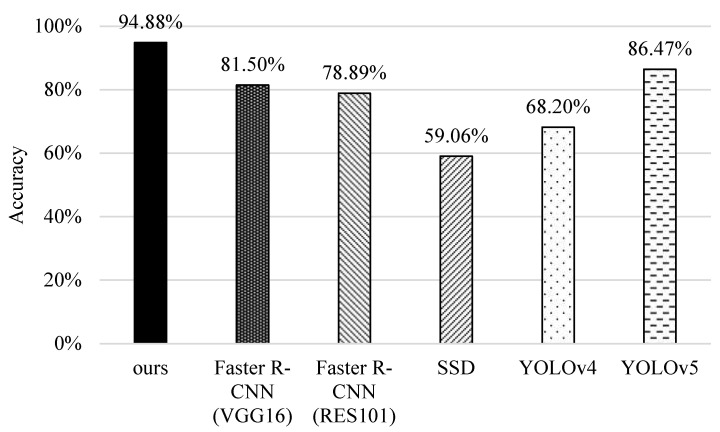
Comparisons of accuracy of different methods.

Finally, we investigate the performance of different methods in terms of accuracy. As shown in Figure 16, SSD and YOLOv4 perform the worst because they find it difficult to identify small features, and even fail to capture the bounding boxes when fish are flipping over or shading with each other. The original faster R-CNNs perform better. Note that VGG16 performs slightly better than RES101 because it removes pooling operation after the first convolutional layer, and thus the feature retention is better. YOLOv5 performs better than them because it substantially improves the detection capability and leverages new estimation functions to reduce the identification error. Our scheme performs the best, mainly because of the prioritizing categories and coherent image analysis, which greatly improve the identification of fish in multiple categories.

### 5.5. Identification of Unknown Data Lists

**Figure 17:**
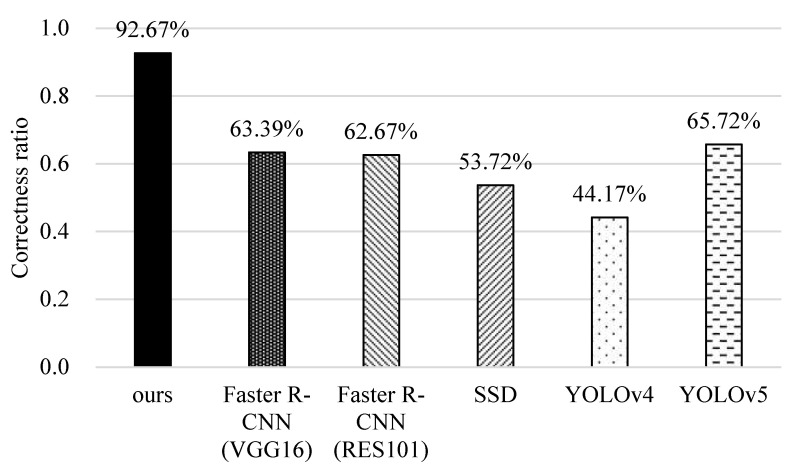
Comparisons of correctness ratios of different methods.

Here, we discuss the average correctness ratio of different methods in identifying the unknown data list <*B_x_*, *I_x_, S_x_>*. Here, the unknown list contains three items: the unknown list of fish bounding box coordinates (*B_x_*), unknown fish-IDs (*I_x_*), and unknown physiological states (*S_x_*). The ratio is calculated based on the three items for each fish. As shown in Figure 17, YOLOv4 and SSD perform the worst because they find it difficult to distinguish between rotten-tail and healthy fish, and even fail to identify their bounding boxes and IDs. YOLOv5 and the original faster R-CNNs (i.e., VGG16 and RES101) perform better because YOLOv5 significantly improves the identification capability and the original faster R-CNNs use a two-stage for object detection to reduce misidentification. However, they still assign many wrong fish-IDs. Our method is the best, mainly because it can effectively identify the fish-ID through the tracking mechanism. Even if the bounding boxes of fish disappear, it can trace them back from the previous time and perform ID correction periodically. Thus, it can improve the overall correctness ratio.

### 5.6. Computational Complexity

**Figure 18:**
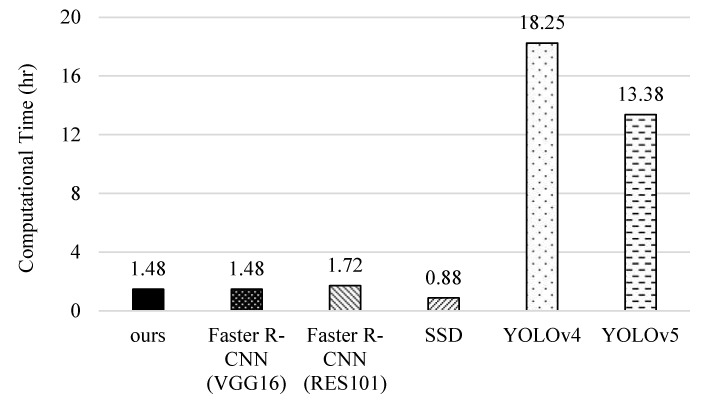
Comparison of the computational complexity of different methods.

Finally, we compare the computational complexity of different methods. The computation time for data training is measured by the platform of ASUS D320MT with Intel Core i5-6400 CPU@ 2.7GHz, 8GB RAM, and NVIDIA GeForce^®^ RTX 2080 8GB Graphic Card (Intel and Nvidia, Santa Clara, CA, USA). In Figure 18, we can see that YOLOv4 takes the longest time because its networks are more complicated and it needs to use a proper batch-size to improve recognition accuracy. Contrarily, YOLOv5 resizes the network model and improves the estimation process to reduce the training time. For the original faster R-CNNs, RES101 takes more time than VGG16 because it performs a pooling operation after the first convolutional layer. Although our scheme needs more time than SSD, it can achieve higher precision, recall, F1-score, accuracy, and correctness ratio (referring to Figure 13, Figure 14, Figure 15, Figure 16 and Figure 17).

## 6. Conclusions

In this paper, we have addressed the misidentification problem of fish physiological state owing to fish swimming behaviors, such as grouping, shading, and flipping over. We took guppy as a case study and focused on the three common physiological states of fish, including healthy, diseased (rotten-tail), and dead. We have proposed a three-phase detection scheme by combining image recognition with the concept of multi-object tracking. Specifically, the first phase is to obtain high-quality data through the specific image pre-processing, including fish data acquisition, data augmentation, and feature selection. In addition, we enhanced the image recognition capability of small feature identification by integrating the prioritizing rules into the image recognition model. In the second phase, we designed a fish-ID tracking mechanism by calculating the predefined similarity functions based on the photograph and trajectory of bounding boxes, and then analyzing the physiological state of the tracked fish through coherent frames, thus achieving more accurate identification. In the third phase, we developed a fish-ID correction mechanism to effectively detect the wrong IDs by comparing a temporary database and activate the correction mechanism timely. Thus, this can ensure the correctness of the tracking results, which can potentially maintain higher accuracy. Through the experimental results, we have verified that our scheme has better performance in fish physiological state identification. The accuracy reaches up to 94.9%, which is about 13.3~15.9% better than that of the original faster R-CNNs. This is because our scheme leverages prioritizing categories to improve the instant recognition capability and analyzes coherent images based on the tracking results, which can potentially avoid misidentification.

## Figures and Tables

**Table 1:** An example of a labeled and unknown data list.

***Labeled Data List*** *<B_f_, I_f_, S_f_>*	
*B_1_*	*((x_1_, y_1_, * *l_1_, w_1_), (x_2_, y_2_, * *l_2_, w_2_), (x_3_, y_3_, * *l_3_, w_3_), (x_4_, y_4_, * *l_4_, w_4_), (x_5_, y_5_, * *l_5_, w_5_), …)*
*I_1_*	*(Nemo, Bob, Mary, Alice, Tom…)*
*S_1_*	*(H, R, R, D, H, …)*
*B_2_*	*((x_1_, y_1_, * *l_1_, w_1_), (x_2_, y_2_, * *l_2_, w_2_), (x_3_, y_3_, * *l_3_, w_3_), (x_4_, y_4_, * *l_4_, w_4_), (x_5_, y_5_, * *l_5_, w_5_), …)*
*I_2_*	*(Bob, Mary, Alice, Tom, Nemo…)*
*S_2_*	*(R, R, D, H, H …)*
*B_3_*	*((x_1_, y_1_, * *l_1_, w_1_), (x_2_, y_2_, * *l_2_, w_2_), (x_3_, y_3_, * *l_3_, w_3_), (x_4_, y_4_, * *l_4_, w_4_), (x_5_, y_5_, * *l_5_, w_5_), …)*
*I_3_*	*(Mary, Nemo, Bob, Alice, Tom…)*
*S_3_*	*(R, H, R, D, H…)*
*B_4_*	*((x_1_, y_1_, * *l_1_, w_1_), (x_2_, y_2_, * *l_2_, w_2_), (x_3_, y_3_, * *l_3_, w_3_), (x_4_, y_4_, * *l_4_, w_4_), (x_5_, y_5_, * *l_5_, w_5_), …)*
*I_4_*	*(Bob, Mary, Alice, Tom, Nemo…)*
*S_4_*	*(R, R, D, H, H…)*
*B_5_*	*((x_1_, y_1_, * *l_1_, w_1_), (x_2_, y_2_, * *l_2_, w_2_), (x_3_, y_3_, * *l_3_, w_3_), (x_4_, y_4_, * *l_4_, w_4_), (x_5_, y_5_, * *l_5_, w_5_), …)*
*I_5_*	*(Tom, Nemo, Bob, Mary, Alice…)*
*S_5_*	*(H, H, R, R, D…)*
*…*	*…*
***Unknown Data List*** *(B_x_, I_x_, S_x_)*	
*B_x_*	*((?, ?, ?, ?), (x_2_, y_2_, w_2_, h_2_), (?,?,?,?), (x_4_, y_4_, w_4_, h_4_), (?,?,?,?), …)*
*I_x_*	*(Bob, ?, Alice, ?, …, …)*
*S_x_*	*(?, R, ?, D, ?, …)*

**Table 2:** Common parameters of the schemes [16,27,33,34].

Parameter	Ours	Faster-RCNNs	SSD	YOLOv4/v5
learning rate	0.001~0.0001	0.001~0.0001	0.001~0.0001	0.01~0.001
batch size	256	256	8	64/16
momentum	0.9	0.9	0.9	0.949/0.98
weight decay	0.0005	0.0005	0.0005	0.0005/0.001
**Parameters of the proposed scheme**				
$({\tilde{t}}_{H},{\tilde{t}}_{R}\;$$,\;{\tilde{t}}_{D}$) = (0.5, 0.3, 0.9)			$\tilde{b}$= 5	
$\tilde{r}$= image length/3			${\tilde{f}}_{t}$= 9	
*Ω* = 1.5			$\tilde{m}$= 20	
$\tilde{d}$= 5			λ = 5	
${\tilde{f}}_{H}$= 4			${\tilde{t}}_{H}$= 0.14	
$\tilde{p}$= 10			α = 0.15	

**Table 3:** Confusion matrix.

Actual Class\Predicted Class	Positive	Negative
Positive	TP	FN
Negative	FP	TN

## Data Availability

The data used to support the findings of this study are included within the article.
